# Application of SPARK teaching in acute abdomen radiography teaching for undergraduate medical students

**DOI:** 10.1186/s12909-022-03957-9

**Published:** 2022-12-19

**Authors:** Yangsheng Li, Chengcheng Gao, Xiangwen Zhu, Jiying Zhu, Zhongxiang Ding, Zhijiang Han

**Affiliations:** Department of Radiology, Affiliated Hangzhou First People’s Hospital, Zhejiang University School of Medicine, 310006 Hangzhou, Zhejiang China

**Keywords:** SPARK teaching mode, Acute abdominal pain, Clinical practice teaching

## Abstract

**Background:**

Acute abdomen is a series of acute and severe abdominal diseases commonly encountered in clinic. It is important to strengthen the image teaching of acute abdomen for undergraduates.

**Aim:**

This study aimed to explore the application effect of SPARK[sub-speciality (S), problem-based learning (P), assessment (A), report (R) and reading skill (K)] teaching mode in the experimental teaching of acute abdomen for undergraduate medical students.

**Methods:**

We selected a total of 58 third year medical students for observation. The students were divided into experimental group and control group. Among them, 29 students in the experimental group studied in SPARK teaching mode, 29 students in the control group studied in traditional teaching mode. The two groups of students were tested after the theory class, before and after the experimental class, and one week after the experimental class, to compare the application effects of the two teaching modes. After the test one week after the experimental class, the two groups of students jointly adopted SPARK mode to learn, and were tested again one month after the experimental class to compare whether the two groups of students achieved the same results. The total score of all tests was 150.

**Results:**

The average scores of the experimental group and the control group after theory class were (69.0 ± 26.4) and (72.1 ± 24.1) respectively, with no statistical difference (*t* = 0.468, *P* = 0.642). The average scores of the experimental group before, after and one week after the experimental class were higher than those of the control group. The experimental group was (84.5 ± 23.1), (109.7 ± 23.8), (105.5 ± 31.0) respectively, and the control group was (52.8 ± 15.1), (93.8 ± 17.0), (80.0 ± 22.8) respectively. The differences were statistically significant (*t* = -6.195, *P* = 0.00; *t* = -2.919, *P* = 0.05; *t* = -3.569, *P* = 0.01). The average scores of the experimental group and the control group after one month were (99.0 ± 31.0) and (95.5 ± 25.6) respectively, and there was no significant difference between the two groups (*t* = -0.462, *P* = 0.646).

**Conclusions:**

The SPARK teaching mode was helpful for undergraduate medical students to consolidate image foundation, improve image reading skills.

## Background

Acute abdomen is a general term for a series of common clinical abdominal diseases. The most common ones include gastrointestinal perforation, obstruction, intussusception, acute pancreatitis, acute appendicitis, rupture and bleeding of parenchymal organs. Patients are often hospitalized with acute abdominal pain, which has the characteristics of acute onset, rapid progression, complex and critical condition [1]. Clinicians need to make accurate diagnosis in the first time. At present, the diagnosis of acute abdomen depends highly on radiological examination [2, 3]. The clinicians' mastery of certain acute abdomen image reading skills is conducive to their first assessment of the patient's condition and to avoid possible subsequent medical risks. Therefore, the teaching of imaging knowledge related to acute abdomen diseases should be strengthened at the initial stage of undergraduate education. Sub-speciality (S), problem-based learning (P), assessment (A), report (R) and reading skill (K) are important links in the imaging teaching of acute abdomen. We summarize them as SPARK teaching mode, which helps students to consolidate their imaging foundation during the undergraduate period and cultivate their comprehensive and systematic understanding of acute abdomen related diseases.

At present, the education of undergraduate medical students in China is still based on the traditional teaching mode. Teachers explain knowledge points according to the teaching syllabus, while students passively receive knowledge in class and review independently through textbooks or teaching courseware after class [4]. This teaching mode is dominated by teachers, while students are in a passive position, and their clinical thinking and practical ability can not be well cultivated, resulting in poor teaching effect. In addition, Chinese national conditions determine that there are a large number of students taking classes at the same time in higher education institutions. At the same time, the arrangement of theoretical courses is short and the effect of multimedia equipment in schools in some underdeveloped areas is not very good. Some students lack the motivation and binding force for learning. These objective and subjective factors make the effect of undergraduate image teaching impossible to be guaranteed. Furthermore, due to the outbreak of COVID-19 in recent years, online teaching mode gradually plays an important role in order to avoid large-scale infection caused by students' offline classes [5–8]. Because of the above reasons, the reform of image teaching mode is imperative.

In recent years, a variety of new teaching models have been successively launched in medical education [9, 10], such as problem-based learning(PBL), case-based learning(CBL) and so on, and have achieved satisfactory results [11–14]. In CBL teaching method, teachers usually provide real clinical cases, and students analyze and discuss the cases according to the theoretical knowledge they have learned [15, 16]. PBL teaching method introduces problems into cases, and students consult materials and discuss in groups to solve problems [17, 18]. The former aims to guide students to combine theory with clinical practice, while the latter mainly cultivates students' thinking and ability to analyze and solve problems. Both of the two teaching modes take students as the main body, which improves students' subjective initiative in learning, enables them to have a deeper understanding and mastery of some knowledge points, enlivens the classroom atmosphere to a certain extent, and stimulates students' learning enthusiasm. However, the above-mentioned teaching modes have inevitable shortcomings in undergraduate education. As undergraduate medical students are still in the initial stage of imaging, it is difficult for them to deeply analyze cases and solve problems, which will easily increase their burden. The above two teaching modes are more suitable for people with a certain professional foundation. In addition, undergraduate students need to learn a wide range of basic knowledge. Too detailed teaching mode can not enable them to master all the knowledge required by the syllabus within the limited class time. If the advantages of the above teaching mode can be absorbed, students' learning can be transformed into directive task, and assessment indicators can be set up to quantify the learning effect, that is, the so-called "task-based teaching mode" can better improve the teaching effect of undergraduate medical students.

We developed a corresponding software platform based on SPARK teaching model, which combines the clinical needs and the advantages of the above new teaching model, and provides a new method for undergraduate imaging teaching. Students can install related software on mobile devices, and learn repeatedly anytime and anywhere. Teachers can observe students' learning progress and evaluate their learning effect in real time in the software background. In this study, we introduced SPARK teaching mode into the experimental teaching of acute abdomen for undergraduate medical students, and compared the teaching effect with the traditional mode, so as to evaluate the teaching effect of SPARK teaching mode, in order to provide new methods and ideas for the reform of image teaching mode.

## Methods

### Participants

This study was a prospective controlled experiment. A total of 58 third year medical students majoring in clinical medicine in the Fourth Clinical Medical College of Zhejiang University of traditional Chinese medicine, who would offer medical imaging courses in the second semester of 2021–2022, were recruited participate. ALL students were informed and volunteered to participate in the study. According to the curriculum arrangement, all students were divided into experimental group and control group.There were 29 subjects in the control group, including 10 males and 19 females, aged 21–23, with an average age of (21.2 ± 0.5) years. There were 29 subjects in the experimental group, including 13 males and 16 females, aged 21–22 years, with an average age of (21.2 ± 0.4) years. The gender of the two groups of students (*X*^*2*^ = 0.648, *P* = 0.421) and age (*t* = 0.581, *P* = 0.564) were comparable.

### Inclusion and exclusion criteria

Inclusion criteria: ① Participate in the teaching of acute abdomen theory course and experimental course; ② Complete all the test questions within the specified time; ③ The students have good compliance, and the completion rate of SPARK test is over 90%. The inclusion criteria ①, ②, ③ were used in the experimental group, and ①, ② were used in the control group; Exclusion criteria: ① Absent from either acute abdomen theory course or experimental course or both; ② Do not complete all the test questions within the specified time; ③ Actively withdraw from the experimental group or the control group; ④ Students' compliance is poor, and the completion rate of SPARK test do not reach 90%. Exclusion criteria ①, ②, ③, ④ were used in the experimental group, and ①, ②, ③ were used in the control group.

### Study design

According to the school's curriculum arrangement, the two groups of students began to study the theory of acute abdomen at the same time in the second semester of 2021–2022, with a total of 2 class hours. The teaching contents include gastrointestinal perforation, obstruction, intussusception, acute pancreatitis, acute appendicitis, rupture and bleeding of parenchymal organs. After the theory class, all the students were tested at the same time. The average test scores of the two groups of students had no statistical difference and were comparable(*t* = 0.468, *P* = 0.642).

The experimental group studied with SPARK teaching mode. We assigned each student acute abdomen question group before and after the experimental class through the relevant software platform, and the study time was one week respectively. The question group included the following five aspects: ① Sub-speciality(S): Students watched an online seminar on acute abdomen (about 50 minutes) given by a senior imaging doctor. The lecture content included the imaging manifestations of normal abdomen and the pathogenesis, clinical manifestations and imaging manifestations of the above diseases related to acute abdomen; ② Problem-based method(P): There were one short answer questions for each disease of acute abdomen. The problem setting was as follows, please describe the pathogenesis, clinical manifestations, pathological basis, imaging manifestations and differential diagnosis of gastrointestinal perforation. Students needed to consult books and input answers by voice with an accuracy rate of 80%; ③ Assessment(A): In this link, 200 questions were set to interpret the best imaging results of acute abdomen. Students needed to choose the best option according to the pictures. All questions were single choice and there was no time limit for this link; ④ Report(R): There were 50 writing questions for the acute abdomen report. The system set the key words in the report as the drop-down box option, and students could complete the answer only after they selected the correct answer. There was no time limit for this link; ⑤ Reading skill(K): Each acute abdomen disease was set with 1–4 short videos, lasting about 2–3 minutes. Each video was a real clinical case, mainly interpreting the imaging data of the patient, and introducing the general information, history of present disease, physical examination, laboratory examination and prognosis of the patient. The number of questions in the question group before and after the experiment was the same, and the content of the question group after the experiment was adjusted according to the two test results of the experiment. During the period of using SPARK, teachers observed students' learning progress and urged their completion rate to be over 90%.

The control group was taught in the traditional teaching mode. The teacher assigned tasks orally before and after the experimental class, and the students learned by using chapters related to acute abdomen in textbooks and teaching courseware. The learning time was also one week each. During this period, teachers urged students to study through the Wechat group(Wechat is a widely used app in China, mainly for daily conversations, and can send text, pictures and voice messages).

One week after the theory class, the teacher gave experimental classes for the two groups of students, with a total of 4 class hours. The two groups of students were tested before the experimental class. The teacher understood the deficiency of the students' knowledge of acute abdomen according to the test results, adjusted the focus of the lecture in time in class, and made up for the deficiency of the students. After class, there would be a quiz again to check whether the students' listening effect was ideal.

One week after the experimental class, we arranged another quiz to test the learning effect of the two groups of students this week. Finally, to ensure the teaching effect, all SPARK test groups were assigned to the two groups of students after the test was completed one week after the experiment class, and the test was conducted again one month after the experiment class to check whether the two groups of students reached an agreement.

### Data evaluation and statistical analysis

**Fig. 1:**
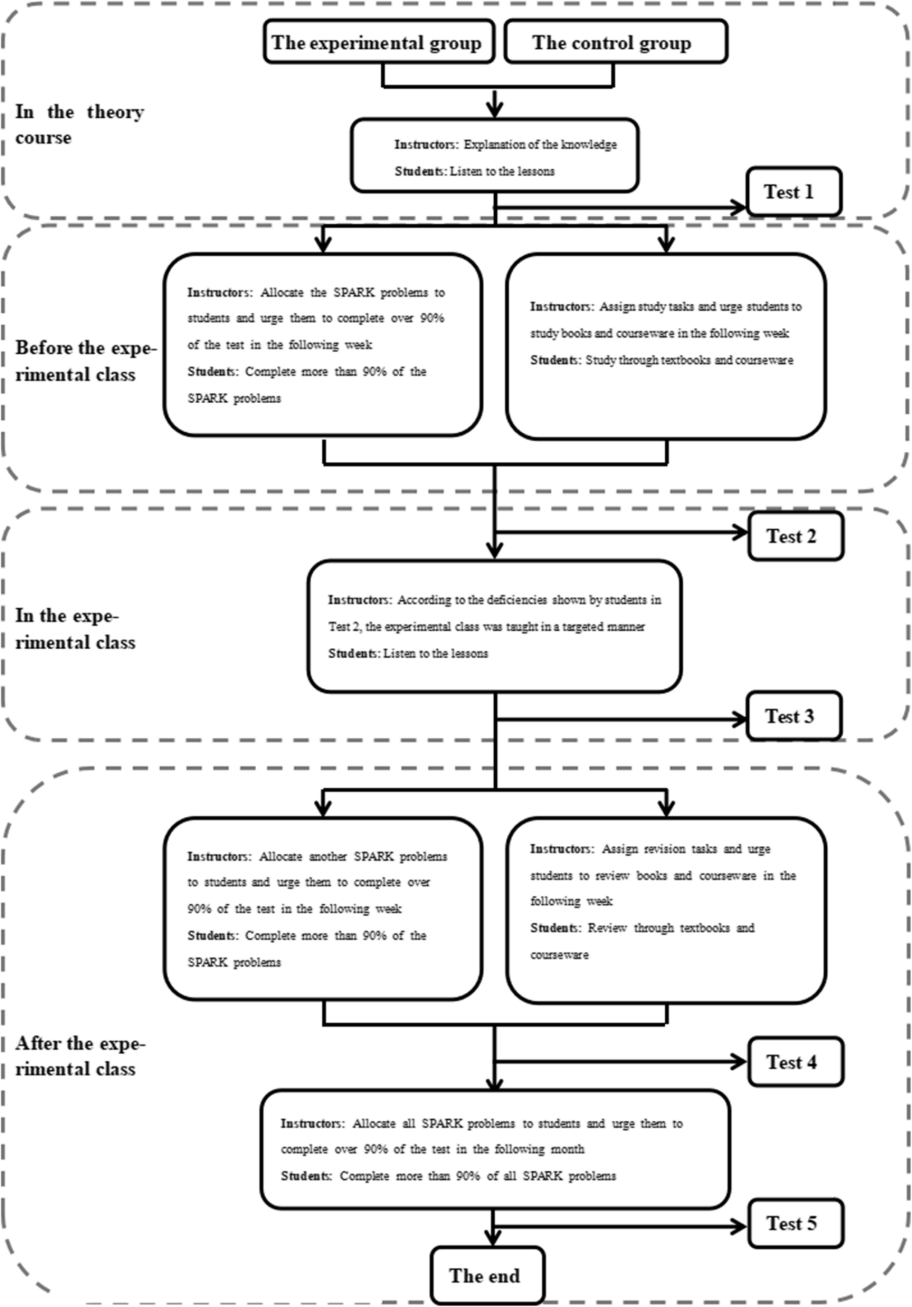
An overview of the study design

**Fig. 2:**
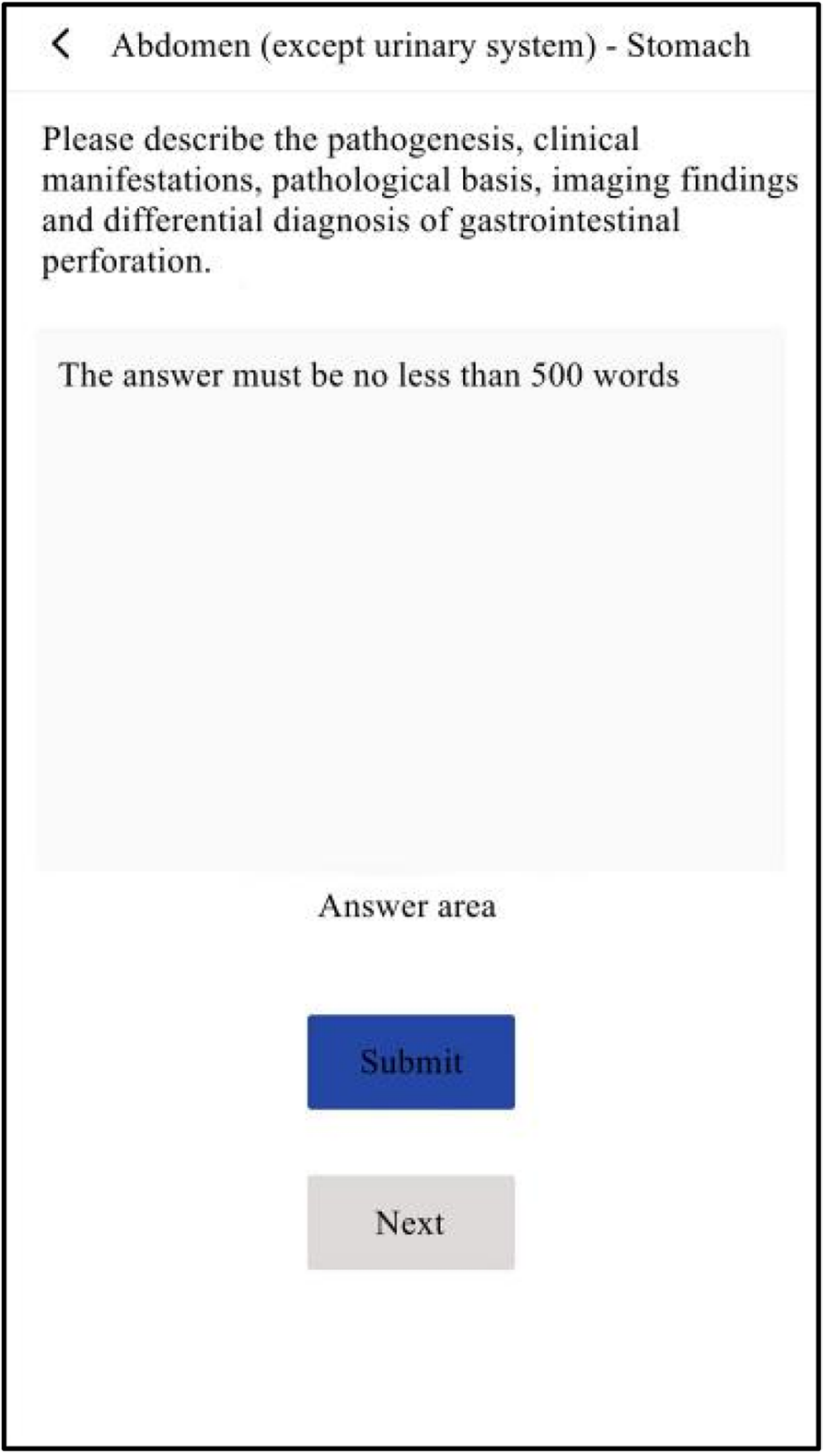
Part of the SPARK schematic diagrams

**Fig. 3:**
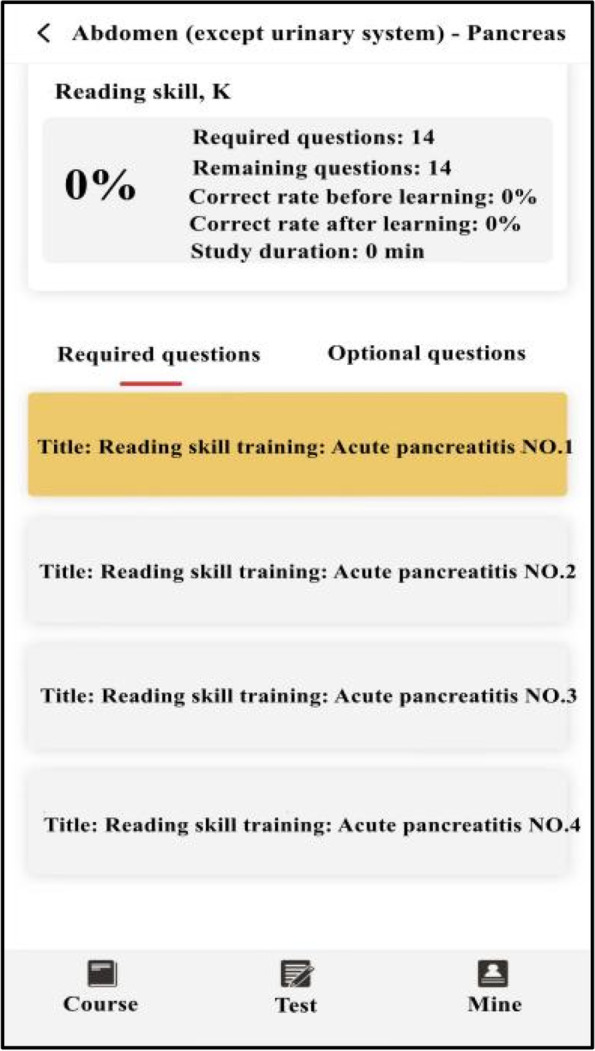
Part of the SPARK schematic diagrams

**Fig. 4:**
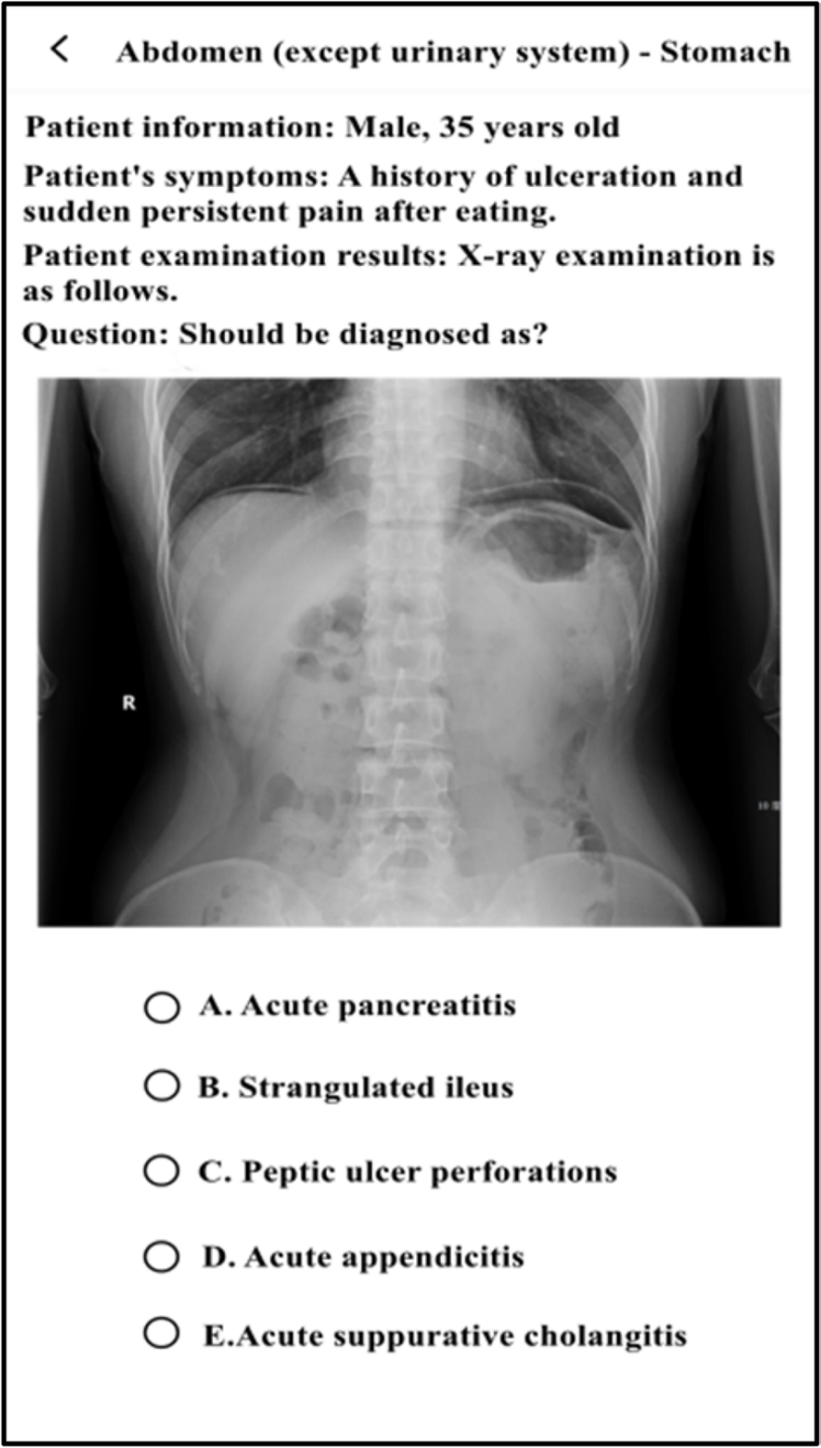
Part of the SPARK schematic diagrams

**Fig. 5:**
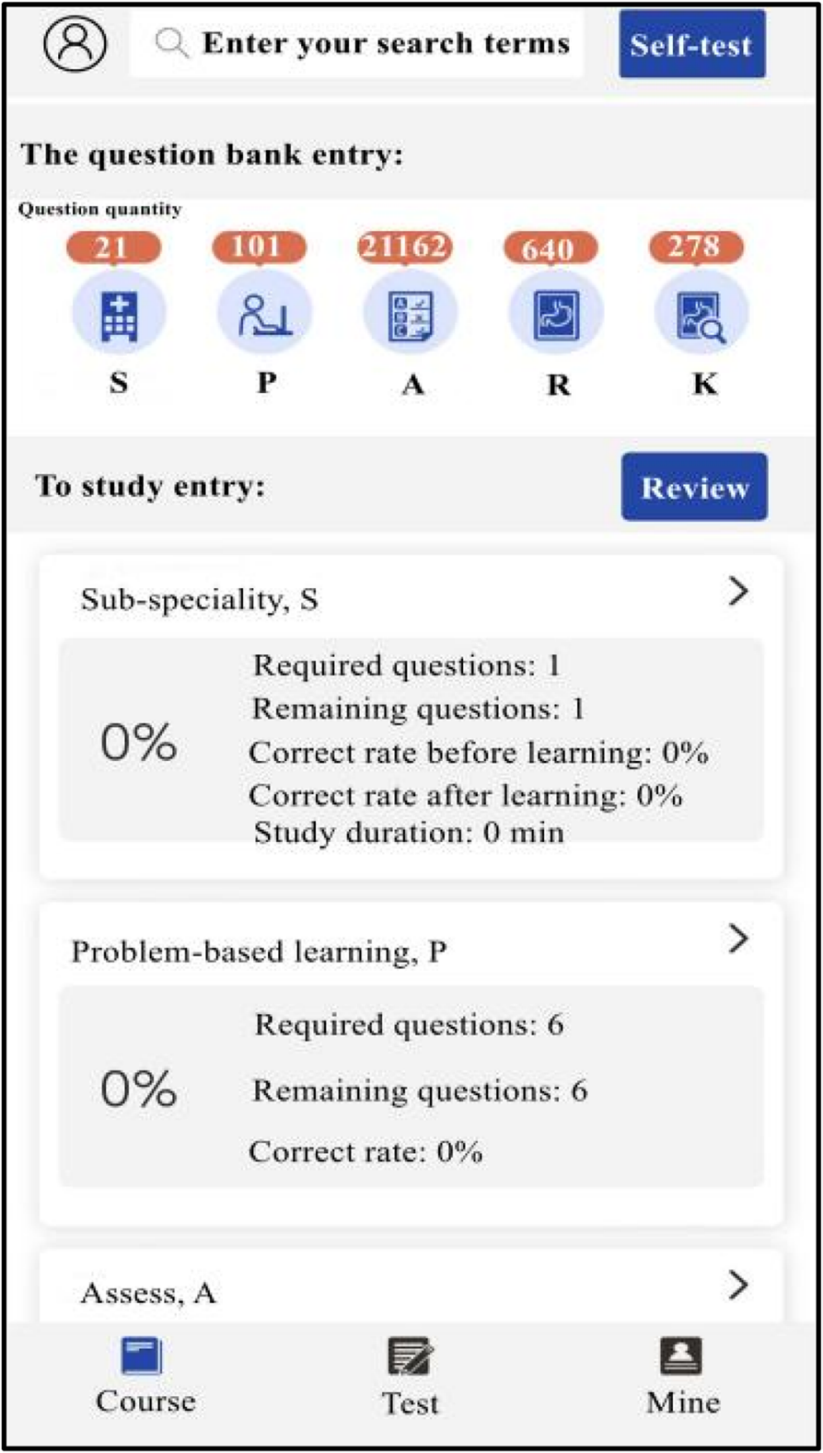
Part of the SPARK schematic diagrams

**Fig. 6:**
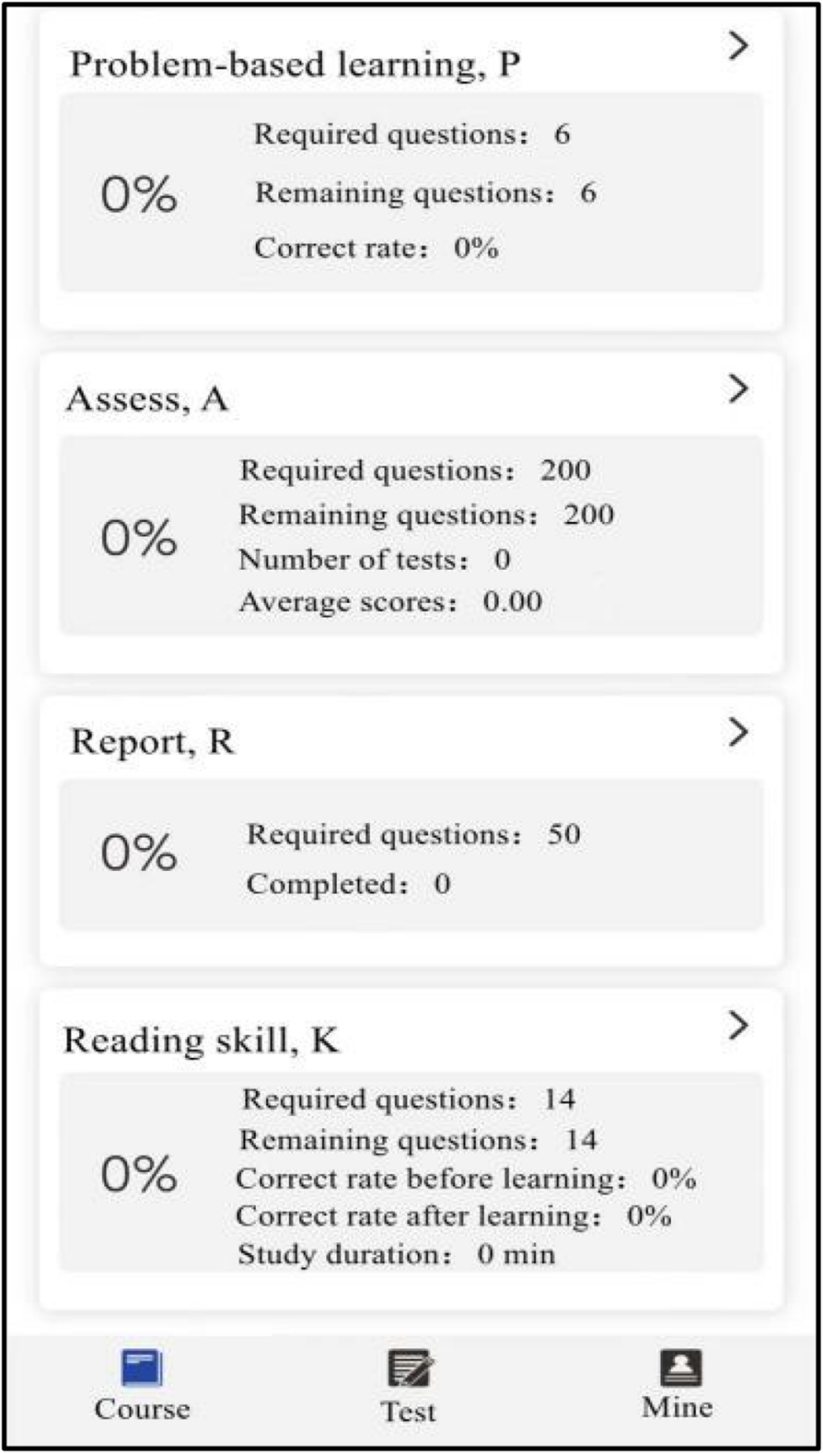
Part of the SPARK schematic diagrams

The two groups of students were given tests after theory class(Test 1), before experiment class(Test 2), after experiment class(Test 3), one week after experiment class(Test 4) and one month after experiment class(Test 5). There were the same test papers before and after experiment class, and the other tests were different. Each test had 15 questions with a total score of 150 points. The two groups of students were required to complete them independently within 10 minutes. The exam questions were set by the third party (senior attending physician) according to the teaching syllabus and were different from those in SPARK. Students could obtain the questions by scanning the QR code on their mobile phones. In order to ensure the reliability and authenticity of the test results, the two groups of students in the same test had the same questions, and the order of questions and options were different. The flow chart of the research design was shown in Fig. 1, and part of the SPARK schematic diagrams were shown in Figs. 2, 3, 4, 5 and 6.

We used SPSS 25.0 statistical software to analyze the data. Kolmogorov-smirnov test was used to test the normality of all scores, and the data of each group followed the normal distribution (*P* > 0.05). Independent sample T-test was used to compare the scores of the two groups to see if there was statistical difference. Measurement data were expressed as (x ± s). Alpha was set at 0.05, *P*-values of less than 0.05 indicated that the difference was statistically significant.

## Results

The attendance rate and completion rate of both theoretical and experimental classes were 100%, while the average completion rates of SPARK in the experimental group were 100%. No one actively withdrew from the experiment, so 29 people in each group were included in the experimental group and the control group, and no one was excluded. There was no statistical difference between the two groups in the average score after theory class, and the range of the lowest and highest scores was basically the same. The average test scores of the experimental group were higher than those of the control group before, after and one week after the experimental class, and the differences were statistically significant. The range of the lowest score and the highest score of the experimental group were also higher than that of the control group. After one month, the test results of the two groups were similar, and the difference was not statistically significant. The range of the lowest score and the highest score was basically the same. See Table 1 for details.

The results before experimental class indicated that the experimental group generally had a poor grasp of plain film and CT images of intestinal obstruction, while most students in the control group had difficulty in identifying acute edema or necrotic pancreatitis. Therefore, the teacher explained the deficiencies of the two groups of students in the experimental class. After the experiment class, the two groups of students' mastery of the corresponding sections were significantly improved.

**Table 1:** Comparison of test scores between the two groups

Group	Cases (n)	Results after theory class	Results before experimental class	Results after experimental class	Results one week after experimental class	Results one month after experimental class
The control group range (mean ± SD values)	29	20–120 (72.1 ± 24.1)	20–80 (52.8 ± 15.1)	40–120 (93.8 ± 17.0)	30–120 (80.0 ± 22.8)	30–150 (95.5 ± 25.6)
The experimental group range (mean ± SD values)	29	30–130 (69.0 ± 26.4)	30–130 (84.5 ± 23.1)	60–140 (109.7 ± 23.8)	30–150 (105.5 ± 31.0)	40–150 (99.0 ± 31.0)
*t* value	-	0.468	-6.195	-2.919	-3.569	-0.462
*P* values	-	0.642	0.000	0.005	0.001	0.646

## Discussion

In this study, in order to compare the application effect of SPARK teaching mode and traditional teaching mode in the experimental teaching of acute abdomen for undergraduate medical students, the students under the two modes were tested for many times and their scores were analyzed. The results showed that the average scores of the students under SPARK teaching mode were higher than those under the traditional teaching mode. Finally, in order to achieve the purpose of high quality and homogeneity of teaching, the scores of the two groups of students reached equilibrium after the intervention of the experimenter.

At present, a single teaching method can no longer meet the learning needs of students in the new era. The hybrid teaching mode has been gradually applied and achieved great results. For example, Men [19] showed that PBL combined with evidence-based learning (EBL) could improve the enthusiasm and initiative of thoracic surgery students and achieve satisfactory teaching effects. Wang [20] studied how to improve the learning effect of ophthalmology graduate students in the teaching of eye trauma, and finally believed that the method of PBL combined with Flipped Classroom(FC) would be more helpful to students without clinical experience. Hu [21] also affirmed the teaching effect of the combination of above two methods, but he believed that further optimization was needed for wider promotion. Xu [22] believed that the combination of mind mapping learning method and PBL teaching could improve students' comprehensive clinical ability better than the traditional learning model. Spark teaching mode is an innovative way opened up by our department when exploring the reform of image teaching mode. It is a new mixed teaching mode based on the advantages of the above teaching mode. This mode can conduct quantitative assessment for each link, and can tailor corresponding learning programs for students with different foundations according to their own characteristics, so that they can use their learning and better integrate imaging knowledge into clinical work. Students can use the relevant software platform to carry out a large number of repeated exercises for the basic knowledge points of images, make full use of fragment time and improve learning efficiency. In addition, the outbreak of the COVID-19 has put forward new requirements for online image teaching. The SPARK teaching mode can also reduce the epidemic prevention risk caused by the offline gathering of students.

There was no statistical difference in the scores after theory class of the two groups of students, that was, after the two groups of students were taught by the same teacher in the same time, their learning effects were basically the same. This result proved that the two groups of students had little difference in radiology imaging basis before the experiment, and their subjective initiative, self-constraint and ability to digest and absorb knowledge in a short period of time were roughly similar, which made the subsequent experiment feasible. After one week of SPARK mode learning, the scores of the experimental group were significantly higher than those of the control group in the traditional learning mode, and the difference was statistically significant. The author analyzes the reasons for the difference as follows: (1) Students in the experimental group could use SPARK software platform to practice repeatedly to find their own weak points, so as to make follow-up learning more targeted, and make full use of the fragmented time to improve learning efficiency. The new teaching mode also greatly inspired students' learning enthusiasm and potential. (2) The S and P links further consolidated the students' image theory foundations, making the boring textbook knowledge lively and interesting. The A and R links provided students with the opportunity to practice and integrated the basic theory and clinical practice more closely. The K link combined clinical data and laboratory examination to systematically sort out image reading ideas for students and improve the level of image diagnosis. The five links complemented each other to break the weak points of students' knowledge one by one and thoroughly achieved the purpose of high-quality teaching. (3) Teachers could use SPARK software platform to monitor students' learning progress, which made the completion rate become a hard indicator to evaluate the learning effect, and played a certain role in supervising students' learning. (4) The solidified traditional teaching mode made the students in the control group lack of interest and motivation in learning, and they were always in the stage of "talking on paper". On the other hand, teachers could not evaluate the students' learning progress and effect in real time, and could not play the important role of restraining students. Therefore, the learning effect of the students in the control group was not as good as that of the experiment.

According to the examination before the experimental class, the teacher targeted to check and fill the gaps for the students, focusing on the weak links of this group of students. Finally, the gaps between the two groups after the experimental class were smaller than those before the experimental class, but the experimental group was still higher than the control group, and the difference was statistically significant. In addition to the scores, the experimental group showed better interaction and cooperation than the control group in class. Finally, in the test after one week of experimental class, the experimental group got better results, the difference was statistically significant, and the difference between the two groups was further increased than that after the experiment. To sum up, we found that the students in the experimental group used SPARK teaching mode for pre-class preparation and post-class review, and achieved satisfactory results, indicating that this teaching mode had wide application and could always run through the whole teaching process of students to help them improve their learning effects in an all-round way.

Different from scientific research experiments, clinical teaching should take students' mastery of knowledge points as the fundamental purpose and provide all students with the same learning opportunities and platforms without discrimination. Therefore, we finally allocated SPARK acute abdomen questions groups to all students. After one month of the experimental class, there was no statistically significant difference between the two groups of students' scores, indicating that all students had basically the same grasp of acute abdomen imaging after learning in SPARK teaching mode. The goal of teaching quality and homogeneity had been achieved.

There were still some shortcomings in this experiment. For example, the sample size was small. When using SPARK teaching platform, some students mechanically completed the task without memorizing the knowledge points by heart. This needs to be solved by formulating a more perfect teaching plan in the future. Secondly, this study was designed as a single center, so it is necessary to conduct a multi-point control study nationwide.

## Conclusion

In general, the SPARK teaching mode had been fully display its advantages in the experimental teaching of acute abdominal. It can help zero based undergraduate medical students consolidate their image foundation, improve their image reading skills, broaden the diagnostic ideas, help students form the image of knowledge system, achieve high-quality teaching purposes, and provide a new method and idea for the reform of image teaching mode.

## Data Availability

The datasets used and analysed during the current study are available from the corresponding author on reasonable request.

## Abbreviations

PBL: Problem-based learning
CBL: Case-based learning
S: Sub-speciality
P: Problem-based learning
A: Assessment
R: Report
K: Reading skill
EBL: Evidence-based learning
FC: Flipped classroom
